# A Novel DNA Repair Gene Signature for Immune Checkpoint Inhibitor-Based Therapy in Gastric Cancer

**DOI:** 10.3389/fcell.2022.893546

**Published:** 2022-05-23

**Authors:** Binbin Yuan, Chengfei Jiang, Lingyan Chen, Lihui Wen, Jinlong Cui, Min Chen, Shu Zhang, Lin Zhou, Yimeng Cai, Jian-Hua Mao, Xiaoping Zou, Bo Hang, Pin Wang

**Affiliations:** ^1^ Department of Gastroenterology, Affiliated Drum Tower Hospital, Medical School of Nanjing University, Nanjing, China; ^2^ Department of Rheumatology and Immunology, The Affiliated Drum Tower Hospital of Nanjing University Medical School, Nanjing, China; ^3^ Berkeley-Nanjing Research Center, Nanjing, China; ^4^ Department of Molecular and Cell Biology, University of California, Berkeley, Berkeley, CA, United States; ^5^ Biological Systems and Engineering Division, Lawrence Berkeley National Laboratory, Berkeley, CA, United States

**Keywords:** gastric cancer, immunotherapy, immune checkpoint blockade, immune checkpoint inhibitors, DNA repair gene signature, prognostic biomarker, score system

## Abstract

Gastric cancer is a heterogeneous group of diseases with only a fraction of patients responding to immunotherapy. The relationships between tumor DNA damage response, patient immune system and immunotherapy have recently attracted attention. Accumulating evidence suggests that DNA repair landscape is a significant factor in driving response to immune checkpoint blockade (ICB) therapy. In this study, to explore new prognostic and predictive biomarkers for gastric cancer patients who are sensitive and responsive to immunotherapies, we developed a novel 15-DNA repair gene signature (DRGS) and its related scoring system and evaluated the efficiency of the DRGS in discriminating different molecular and immune characteristics and therapeutic outcomes of patients with gastric adenocarcinoma, using publicly available datasets. The results demonstrated that DRGS high score patients showed significantly better therapeutic outcomes for ICB compared to DRGS low score patients (*p* < 0.001). Integrated analysis of multi-omics data demonstrated that the patients with high DRGS score were characteristic of high levels of anti-tumor lymphocyte infiltration, tumor mutation burden (TMB) and PD-L1 expression, and these patients exhibited a longer overall survival, as compared to the low-score patients. Results obtained from HPA and IHC supported significant dysregulation of the genes in DRGS in gastric cancer tissues, and a positive correlation in protein expression between DRGS and PD-L1. Therefore, the DRGS scoring system may have implications in tailoring immunotherapy in gastric cancers. A preprint has previously been published (Yuan et al., 2021).

## Introduction

Gastric cancer is one of the most common cancers worldwide, with more than one million new cases in 2020, ranking fifth in incidence and third in mortality worldwide (Abbott et al., 2016). Up to now, the prognosis of this disease at advanced stages remains dismal. Gastric cancer is a heterogeneous group of diseases with variable responsiveness to treatment such as chemotherapy and immunotherapy, and new biomarkers are needed to identify patients with gastric cancer for sensitivity toward such therapies.

Chemotherapy is used as standard treatment for gastric cancer, either as preoperative or postoperative therapy, at all pathological stages of the disease. Some regimens showed better 5-years survival (OS) or relapse-free survival (RFS) rates in certain patients. However, some regimens demonstrated considerable toxicity and mortality in reports (Harada et al., 2018; Joshi and Badgwell, 2021).

In recent years, the approval of multiple immune checkpoint inhibitors (ICIs) and the promising results from clinical trials of tumor immunotherapies have led to the development of tumor immunotherapy (Harada et al., 2018; Joshi and Badgwell, 2021). In ICI therapies, anti-PD-1 and anti-PD-L1 antibodies and anti-CTLA4 antibodies are highly effective in patients with microsatellite instability-high (MSI-H) subtype or high expression of PD-L1 (Chao et al., 2020; Fuchs et al., 2021). Most recently, the effect of immunotherapy combined with chemotherapy or targeted therapy on gastric cancer has been studied. For example, in CheckMate-649, the largest global randomized phase III clinical study in gastric cancer (Moehler et al., 2020), nivolumab plus chemotherapy as first-line treatment for previously untreated and advanced gastric cancer demonstrated superior OS and RFS compared to chemotherapy alone, reducing the risk of death by 20%. Some conflicting results have also been obtained with combined therapy, which may be due to different chemotherapy regimens and heterogeneity of patients. In summary, even though its impact on the outcome of gastric cancer has not been clearly defined, today, immunotherapy plays an important role in the treatment of gastric cancer, and combinational therapy is becoming a new trend.

The association between genetic instability or DNA repair defects with tumor susceptibility to immunotherapy has been observed in certain types of tumors. DNA damage and genomic instability have been found to affect the anti-tumor immune response. There are several major repair pathways for the removal of different types of exogenous and endogenous DNA lesions, including direct reversal, base excision repair (BER), nucleotide excision repair (NER), mismatch repair (MMR) and double-strand break (DSB) repair that includes homologous recombination (HR), non-homologous end joining (NHEJ) and the Fanconi anemia pathway (Hang, 2004; Friedberg et al., 2015; Rodríguez and D'Andrea, 2017). Deficiency in such repair is associated with reduced DNA repair capacity and increased genetic instability, thus promoting cancer development. It can also provide an opportunity or benefit for cancer therapy, as the efficacy of certain anticancer drugs or therapies is highly influenced by cellular DNA repair capacity, for example, small-molecule inhibitors of DNA repair have been combined with conventional chemotherapy drugs (Helleday et al., 2008). As mentioned above, the noticeable efficacy of ICIs in cancers with MMR-deficient and its characteristic genetic signature MSI has recently been discovered, and a large-scale analysis showed that mutations of mismatch repair genes and DNA polymerases account for 13.5% of high tumor mutation burden (TMB) tumors (Chalmers et al., 2017; Le et al., 2017). There is also evidence of potential implications for immunotherapy in cancers with homologous recombination deficiency (HRD) and somatic changes in the NER pathway (Bever and Le, 2018). Taken together, accumulating evidence suggests that a systemic understanding of DNA repair landscape in tumor may help assess the tumor susceptibility to immunotherapy.

There are still many challenges in the application of tumor immunotherapy, for instance, only a small portion of tumors are susceptible, the overall clinical response rate is low and it remains difficult to accurately predict treatment efficacy and response. Such limitations warrant a search for new immunotherapy biomarkers, such as those based on PD-L1 expression, tumor-infiltrating lymphocytes, TMB, deficient MMR, immune gene signatures, and multiplex immunohistochemistry (Gibney et al., 2016). More recently, a role for DNA repair in the selection of patients for immunotherapy has emerged. In this study, we aimed to explore the prognostic role of DNA repair gene expression in gastric cancer in relation to the prediction of response to ICI-based immunotherapy. We identified a 15-DNA repair gene signature (DRGS) and developed a scoring system for clinical utility. In addition, the association of the DRGS score with the molecular and immune profiles was further investigated in the same patients. The results suggest that our DRGS is a promising biomarker for tailoring immunotherapy.

## Materials and Methods

### Datasets Used in This Study

The next-generation sequencing data and clinicopathological information for 407 patients with gastric cancer were downloaded from The Cancer Genome Atlas Stomach Adenocarcinoma (TCGA-STAD) data collection (https://portal.gdc.cancer.gov). The microarray data for 60 patients with gastric cancer in GSE30727 were downloaded from the GEO database (https://www.ncbi.nlm.nih.gov/geo/geo2r/?acc=GSE30727). We also used a dataset with immunotherapy information [the R package “IMvigor210CoreBiologies (version 1.0.0)”] (Balar et al., 2017) to evaluate the prediction efficiency of our scoring system. Drug sensitivity data between different gastric cancer cells *in vitro* were downloaded from Genomics of Drug Sensitivity in Cancer (GDSC, http://www.cancerrxgene.org/downloads).

### Construction of the 15-DNA Repair Gene Signature and Scoring System

From the Human DNA Repair Genes website (https://www.mdanderson.org/documents/Labs/Wood-Laboratory/human-dna-repair-genes.html) (Wood et al., 2005) which was last modified by Drs. R. Wood and M. Lowery on 10th June 2020, a list of 219 DNA repair genes were defined. We used the DEseq2 (version 1.28.1) package in R (version 4.0.5) to analyze the absolute counts for differential gene expression in TCGA-STAD and employed the online platform GEO2R to assess whether these DNA repair genes are differentially expressed between tumor and normal tissues (adjusted *p* < 0.05, |log2FC| > 1) (Supplementary Table S1). Fifteen differentially expressed DNA repair genes were commonly identified in TCGA-STAD and GSE30727, which are: PRKDC, FANCI, LIG1, RECQL4, FANCA, BRCA2, FANCD2, UBE2T, POLQ, PARPBP, EXO1, XRCC2, RAD54L, EME1 and FANCB. These genes constituted our DNA repair gene signature (DRGS). Supplementary Table S2 showed expression level of the 15 genes in the DRGS.

Before the analysis, we applied the Z-score normalization to the expression level of the 15 genes from each patient sample. The principal component analysis (PCA) is a proven technique, which can reduce dimension, improve interpretability and minimize information loss of large datasets at the same time. We employed the PCA as a dimensionality reduction method to obtain the 1st dimension correlation coefficients of the 15 genes (see Supplementary Figure S1 and Supplementary Table S3).

The formula for the DRGS scoring system was as follows:

$$\text{DRGS }\text{score=}{\text{∑}}^{\text{​}}\left(\text{correlation }\;\text{coefficients }\text{for }\text{gene }\text{i }\right)*\left(\text{normalized }\text{gene }\text{i }\text{expression }\text{level }\right)$$ (1)

### Comparison of the Immunocyte Infiltration

To determine the relationship between the DRGS scoring system and immunocyte infiltration in the gastric cancer tissue, we performed immunocyte infiltration analysis through the online platform Immune Cell Abundance Identifier (ImmuCellAI) (http://bioinfo.life.hust.edu.cn/ImmuCellAI#!/) using TCGA-STAD samples (n = 375) (Miao et al., 2020). Then, we calculated DRGS scores for the same TCGA-STAD samples and used the median score as the cut-point to divide these samples into high-score subtype and low-score subtype (Supplementary Table S4). The Mann Whitney U test was used to compare the results of multiple immune cell infiltration between these two subtype groups. We applied hierarchical clustering to immune cells with significantly different infiltration scores between the two groups. The Mann Whitney U test was used to compare the infiltration score between high-score subtype and low-score subtype. We also used ImmuCellAI to predict the patient response to ICB therapy (Supplementary Table S5).

### Comprehensive Analysis of Histologic and Molecular Characteristics

Samples from the two groups mentioned above (TCGA-STAD, High score *n* = 187, Low score *n* = 188) were also used to explore the relationship between the DRGS score and various tumor characteristics. We compared the Lauren classification, molecular subtype and MSI status using the chi-square test (Supplementary Table S6). Tumor mutation levels were calculated through R package “Maftools” (Mayakonda et al., 2018). We used the Mann Whitney U test to compare TMB and PD-L1 expression between the two groups. In light that the TMB and PD-L1 expression are strongly related to the ICI therapy, we performed the Spearman’s correlation to analyze the relationship between the DRGS score and the two immunotherapeutic biomarkers (Supplementary Table S7).

### Relationship Between the 15-DNA Repair Gene Signature Score and Immunotherapy Outcomes

The public dataset IMvigor210CoreBiologies (version 1.0.0) contains a total of 348 advanced urothelial bladder cancer patients who received atezolizumab treatment. We applied the DRGS score to the dataset to investigate its predictive ability of immunotherapy. We performed normalization and PCA analysis with the same method as mentioned above, and all the 348 patients were divided into two equal groups according to their DRGS score (High score *n* = 174, Low score *n* = 174). We then compared their response to immunotherapy and the immunotypes of tumor by the Chi-square test (Supplementary Table S8). Also, the Kaplan-Meier analysis of OS in the two groups was performed, and log-rank test was used to compare survival curves.

### Prognostic Ability of the 15-DNA Repair Gene Signature Score

We also analyzed the prognostic ability of the DRGS scoring system in TCGA-STAD samples, through Kaplan-Meier analysis of OS and the log-rank test for comparison. The independence of the DRGS score system was verified by univariate and multivariate Cox regression. Three independent gastric cancer datasets, GSE26901, GSE15459, and GSE26899, which were downloaded from the GEO database as validation sets and analyzed with the same method described above.

### Association of the 15-DNA Repair Gene Signature Score With Drug Sensitivity

We downloaded the drug sensitivity data from GDSC combined with the RNA-seq data from 23 types of gastric cancer cells. We performed the Pearson correlation analysis to calculate the correlation between the drug sensitivity level of different chemotherapeutic agents and the DRGS score. The criteria for significant correlation were *p* < 0.05 and correlation > 0.4.

### Immunohistochemistry Analysis

After obtaining the consent of 10 gastric cancer patients, tissue sections were obtained from the Department of Pathology, the Affiliated Drum Tower Hospital of Nanjing University Medical School. After blocking with endogenous peroxide and protein, the sections were then incubated with diluted specific PD-L1 and DRGS protein antibodies at 4°C overnight, respectively. The next day, the sections were incubated with a secondary antibody at 37°C for 1 h. The sections were then stained with 3,3-diaminobenzidine (DAB) solution for 3 min and counterstained with hematoxylin, and photographed under a microscope. Two experienced pathologists scored these samples according to the percentage of staining intensity cells and staining intensity score (1 = low positive; 2 = positive; 3 = high positive). The formula for the IHC score was as follows:

$$\text{IHC }\text{score=}{\text{∑}}^{\text{​}}\left(\text{percentage }\text{of }\text{staining }\text{intensity }\text{cells }\right)*\left(\text{staining }\text{intensity }\text{score }\right).$$ (2)

### Verification of Prognosis-Related 15-DNA Repair Gene Signature and Programmed Cell Death Ligand 1 Expression

Data from our IHC results and the Human Protein Atlas (HPA) (https://www.proteinatlas.org/) were analyzed to verify the protein expression of DRGS and PD-L1 in tumor tissues and normal tissues, and to determine whether the expression differences and the correlation between DRGS and PD-L1 were consistent with the previous mRNA results from TCGA. (We performed the Pearson correlation analysis to calculate the correlation between protein expression of DRGS and PD-L1, and the difference was considered significant if the *p*-value is < 0.05.)

### Statistical Analysis

Statistical methods were described in different sections above. The significance level of these statistical tests was two-sided *p* value < 0.05. The software used for analysis is R (version 4.0.5).

## Results

### Identification of a 15-DNA Repair Gene Signature and Construction of the Scoring System

**FIGURE 1:**
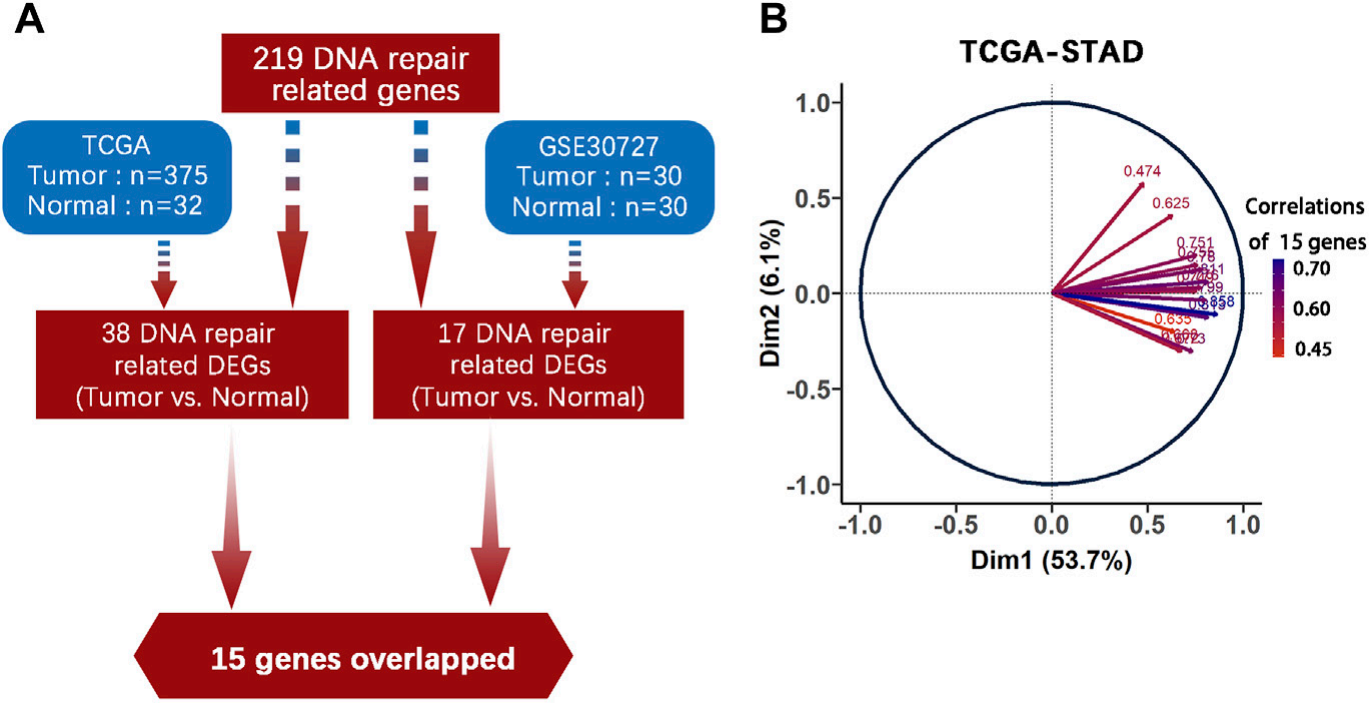
DNA repair genes and construction of the DRGS scoring system. **(A)** Identification of 15 common genes among the 219 DNA repair genes that are differentially expressed in both TCGA-STAD (Tumor *n* = 375, Normal *n* = 32) and GSE30727 (Tumor *n* = 30, Normal *n* = 30); **(B)** PCA analysis was used as a dimensionality reduction method to obtain the 1st dimension correlation coefficients of the 15 repair genes. DEGs: Differentially expressed genes.

219 DNA repair genes were obtained from the Human DNA Repair Genes website as described above. Next, we used DEseq2 package in R to process the raw data from TCGA-STAD and used the online platform GEO2R to find genes differentially expressed between tumor (*n* = 375) and normal (*n* = 32) tissues (adjusted *p* value < 0.05, |log2FC| > 1). Finally, we identified 15 common genes among 219 DNA repair gene, which were differentially expressed in both TCGA-STAD and GSE30727 (Tumor *n* = 30, Normal *n* = 30) (Figure 1A, Table 1). We then used PCA analysis as a dimensionality reduction method to obtain the first dimensional correlation coefficients of the 15 genes (Figure 1B). Based on these coefficients, we created a 15-DNA repair gene signature (DRGS) scoring system (for details, please refer to the methods section). The DRGS score was then used to divide patients into two groups (high and low score groups).

**TABLE 1:** The 15 DNA repair genes in the DRGS and their respective values.

Gene symbol	TCGA-STAD		GSE30727	
	logFC	adj.P.Val	logFC	adj.P.Val
PRKDC	1.081	1.71E-16	1.042	9.93E-04
FANCI	1.825	1.40E-31	1.012	3.08E-05
LIG1	1.012	4.80E-13	1.009	1.70E-02
RECQL4	2.112	5.64E-28	1.007	2.28E-03
FANCA	1.647	5.54E-23	1.412	1.19E-05
BRCA2	1.740	2.90E-22	1.091	3.30E-03
FANCD2	1.582	3.12E-25	1.196	1.04E-03
UBE2T	1.618	2.96E-21	1.036	1.01E-03
POLQ	1.906	5.96E-22	1.182	8.10E-04
PARPBP	1.386	4.54E-18	1.255	2.17E-03
EXO1	2.152	3.02E-32	1.136	6.97E-03
XRCC2	2.177	5.83E-37	1.016	1.33E-03
RAD54L	1.735	4.55E-23	1.290	4.36E-04
EME1	2.160	5.61E-32	1.030	1.05E-03
FANCB	1.789	9.37E-25	1.057	9.79E-04

### Immune Characteristics of Different 15-DNA Repair Gene Signature Score Groups

**FIGURE 2:**
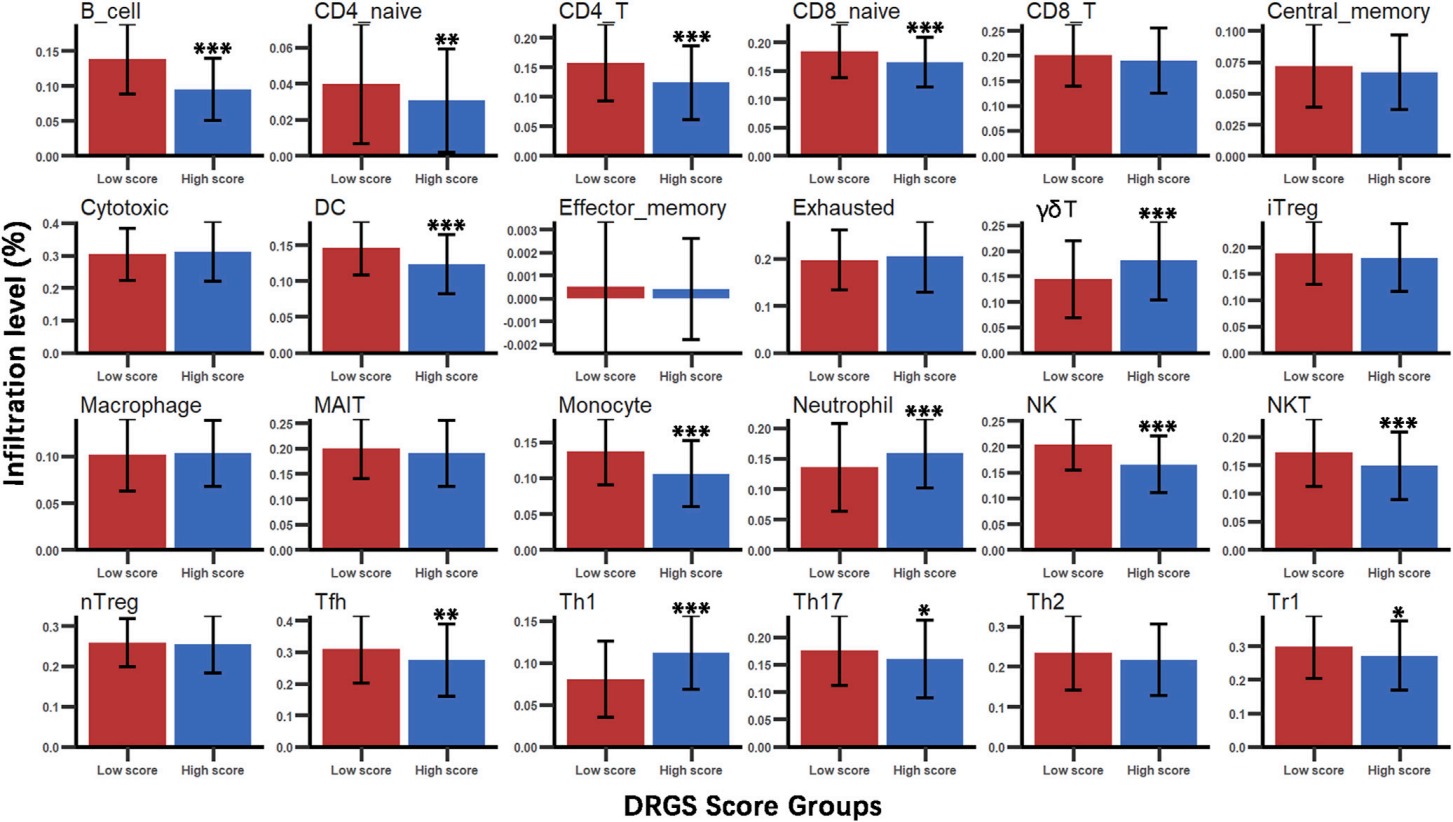
Immune cell types infiltration in DRGS high (*n* = 187) and low (*n* = 188) score subgroups. Mann Whitney U test was used to compare the distribution of multiple immune cell infiltrations between the two groups (*p < 0.05, ** *p* < 0.01, *** *p* < 0.001).

**FIGURE 3:**
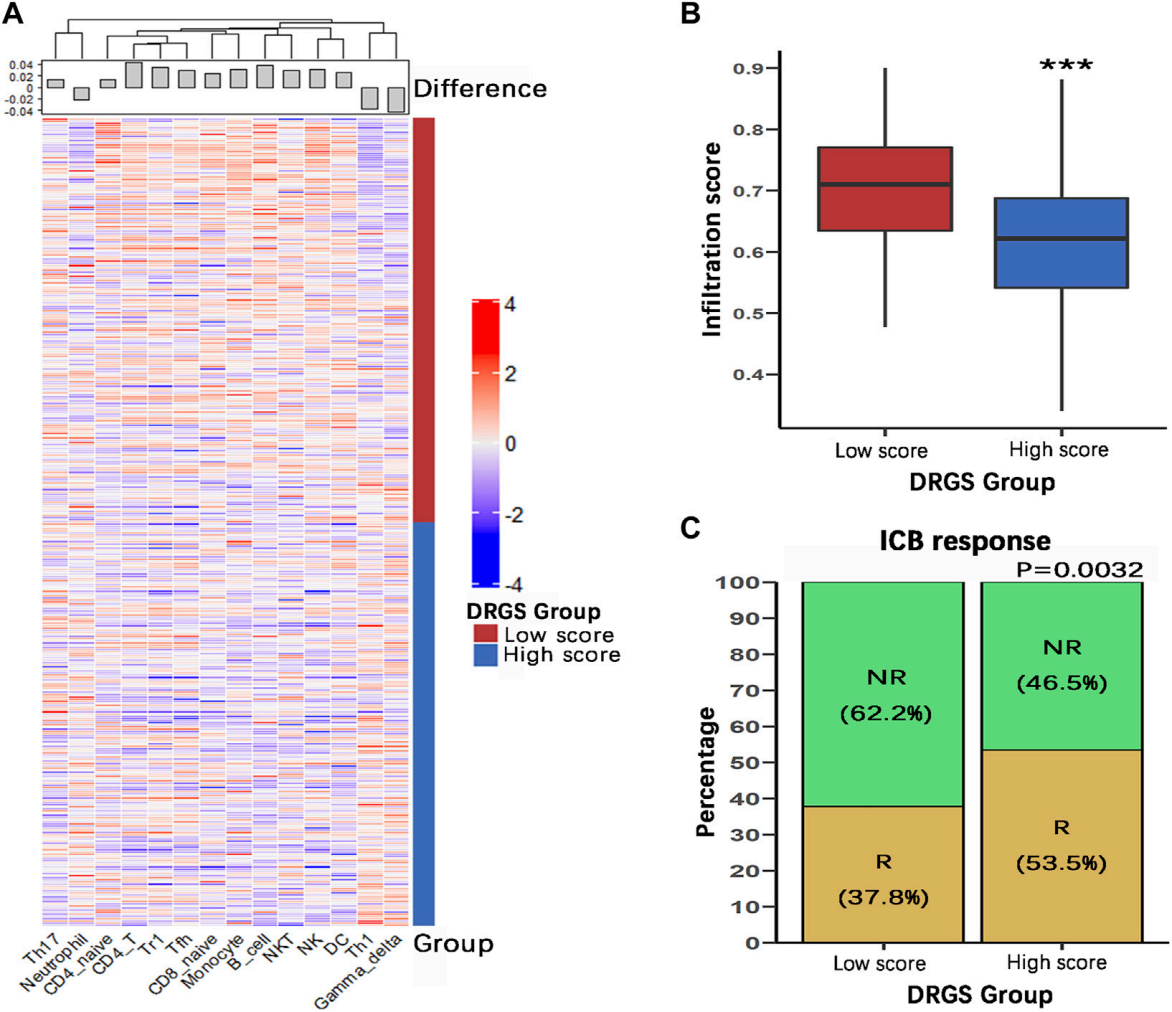
Immune characteristics of the DRGS score subgroups (High score n = 187, Low score *n* = 188). **(A)** Hierarchical clustering of various types of immune cells with significant differences in infiltration scores between the two score groups; **(B)** The overall infiltration score of low score group was significantly higher than the high score group; **(C)** ImmuCellAI was used to predict the response of ICB therapy. R: response; NR: no response. (*p < 0.05, ** *p* < 0.01, *** *p* < 0.001).

We identified the immune characteristics of 375 TCGA-STAD samples through ImmuCellAI to estimate the specific fractions of 24 types of immune cells in each gastric cancer sample (Supplementary Table S4). To analyze the composition of immune cells in different score subgroups (High score *n* = 187, Low score *n* = 188), we used the Mann Whitney U test to compare the distribution of immune cell types in the two score groups. The result showed significant differences in the infiltration rates of 14 immune cells between the two groups. We found that anti-tumor cells such as γδ T cells, neutrophil cells and Th1 cells were more abundant in the DRGS high score group (*p* < 0.001), while immunosuppressive or tumor enhancement cells such as B cells (*p* < 0.001), CD4 T cells (*p* < 0.001), monocytes (*p* < 0.001), Th17 cells (*p* = 0.031), Tfh cells (*p* = 0.002) and Tr1 cells (*p* = 0.012) were more abundant in the DRGS low score group (Figure 2, Figure 3A). In our study, infiltration score of samples in the DRGS low score group was significantly higher than those in the high score group (*p* < 0.001, Mann-Whitney U test, Figure 3B). Furthermore, we used ImmuCellAI to predict the response of ICB therapy (Supplementary Table S5) and found that there would be 53.5% of patients in the DRGS high score group and fewer patients (37.8%) in the DRGS low score group, in response to ICB therapy (*p* = 0.003, Chi-square test, Figure 3C).

### Relationship Between 15-DNA Repair Gene Signature Score Groups and Other Histologic and Molecular Classifications

**FIGURE 4:**
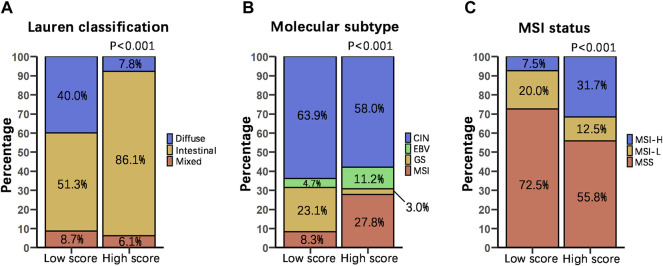
Relationship between score grouping and other histologic and molecular classifications. We compared the percentage distribution of the Lauren classification (High score *n* = 115, Low score *n* = 115) **(A)**, molecular subtype (High score *n* = 169, Low score *n* = 169) **(B)**, and MSI status proportion (High score *n* = 120, Low score *n* = 120) **(C)** of patients with DRGS high and low score using Chi-square test. In all cases, p is less than 0.001.

To examine the relationship between our score grouping and other histologic and molecular classifications, we analyzed clinical data of 372 samples of gastric cancer patients from the TCGA-STAD database (Supplementary Table S6). According to the Lauren classification, gastric cancers can be divided into 3 subtypes, i.e., intestinal, diffuse and mixed. In our study, the DRGS high score group (*n* = 115) was characterized by a very high proportion of intestinal subtype samples (86.1%), while diffuse and mixed subtypes samples were only 7.8 and 6.1%, respectively. On the other hand, the DRGS low score group (*n* = 115) is comprised of 51.3% intestinal, 40.0% diffuse and 8.7% mixed samples. Statistically, there were significantly more intestinal and fewer diffuse type gastric cancer samples in the DRGS high score group as compared to the low score group (*p* < 0.001, Chi-square test, Figure 4A).

TCGA proposed 4 subtypes of gastric adenocarcinoma: 1) EBV-positive, 2) microsatellite instability (MSI), 3) genomically stable (GS), and 4) chromosomal instability (CIN) (The Cancer Genome Atlas Research Network, 2014). In our study, the DRGS high score group (*n* = 169) had a higher percentage of EBV-positive (11.2%) and MSI (27.8%) subtype samples. In contrast, the DRGS low score group (*n* = 169) had a higher percentage of GS (23.1%) and CIN (63.9%) subtype samples (*p* < 0.001, Chi-square test, Figure 4B).

On the basis of the frequency of mutations in microsatellite markers, gastric cancer can be classified as MSS, MSI-H, and MSI-L (Rhyu et al., 1994; Yoon et al., 2013). In our study, the percentage of MSI-H subtype patients (31.7%) was higher in the DRGS high score group (*n* = 120) than that in the low score group (7.5%) (*n* = 120). In contrast, there were more MSS subtype patients (72.5%) in the DRGS low score group than in the high score group (55.8%). (*p* < 0.001, Chi-square test, Figure 4C).

### Prognostic Ability of the 15-DNA Repair Gene Signature Score

**FIGURE 5:**
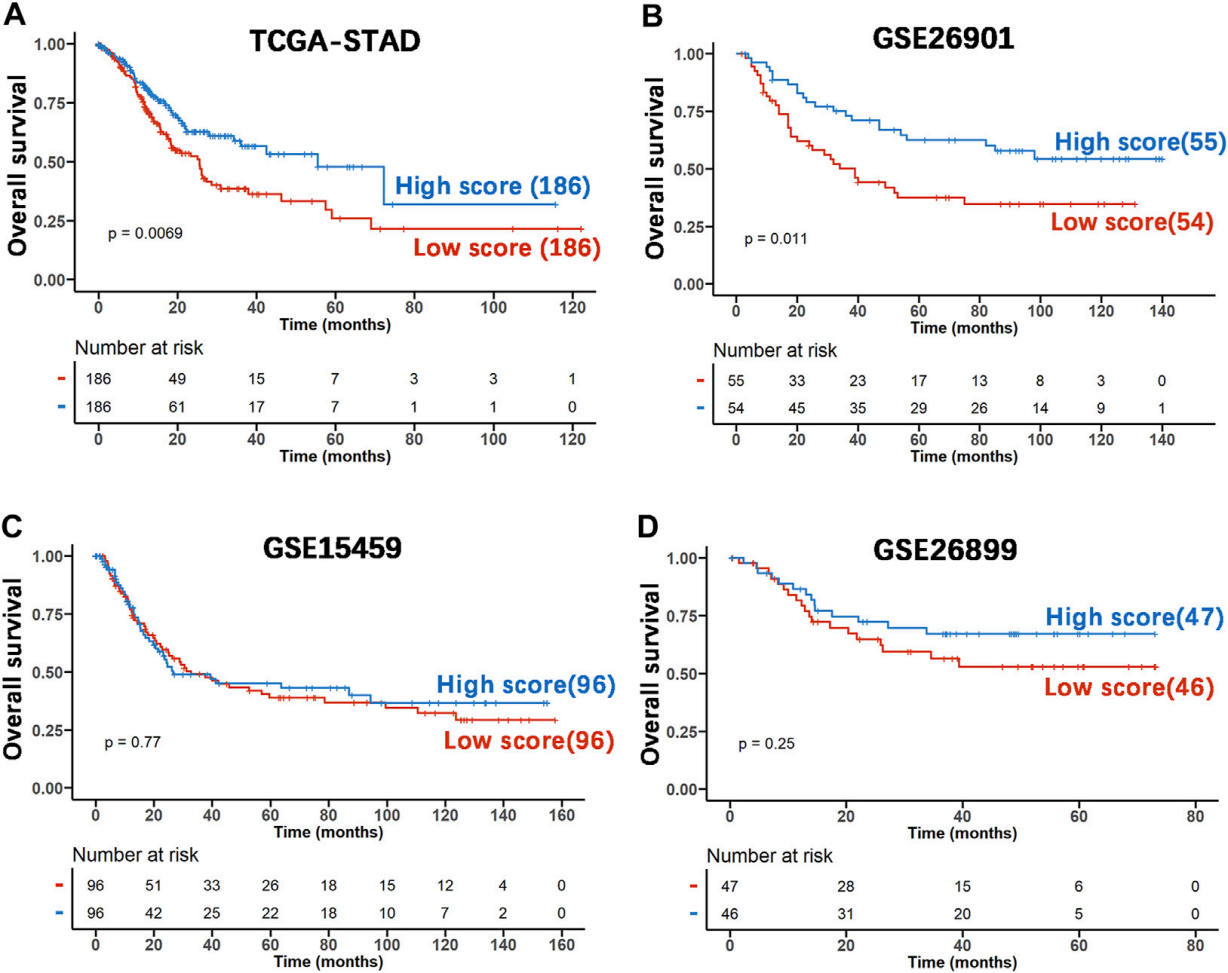
The prognostic capability of the DRGS score system using various publicly available gastric cancer datasets. **(A)** Kaplan-Meier analysis and log-rank test of TCGA-STAD samples were used to compare the OS rate of the two score groups (High score *n* = 186, Low score *n* = 186); **(B)** The independent gastric cancer dataset GSE26901 (High score *n* = 55, Low score *n* = 54) as the validation set was analyzed using the same method as above; **(C)** the dataset GSE15459 (High score *n* = 96, Low score *n* = 96) was used as the validation set; **(D)** GSE26899 (High score *n* = 47, Low score *n* = 46) was used as the validation set.

To investigate whether the prognostic impact of the DRGS score is independent of clinical factors that could be associated with patient outcomes, we first performed univariate Cox regression analysis on all available clinical parameters in the TCGA-STAD dataset (*n* = 372). Clinical parameters that demonstrated significant prognostic impact (*p* < 0.05 by the Wald test) were selected for multivariate Cox regression analysis along with the DRGS. The result indicated that the DRGS is an independent prognostic factor (*p* = 0.038, Table 2). Taking the median score as the cut-off value, patients with DRGS high score had a better OS than those with DRGS low score (*p* = 0.007, log-rank test, Figure 5A). Then, the prognostic value of scoring system was validated using the GSE26901 (*n* = 109), GSE15459 (*n* = 192) and GSE26899 (*n* = 93) gastric cancer datasets. As shown in Figure 5, the patients in GSE26901 in the DRGS high score group had a significantly better prognosis than those in the low score group (*p* = 0.011, log-rank test, Figure 5B), consistent with the results from the TCGA dataset. However, there was no significant OS difference in GSE15459 (*p* = 0.77, Figure 5C) or GSE26899 (*p* = 0.25, Figure 5D) between the two DRGS groups.

**TABLE 2:** Univariate and multivariate Cox regression confirm the independence of the DRGS scoring system.

Variables	HR	95% CI for HR*		*p* values
		Lower	Upper	
Clinical factors				
Age	1.041	1.020	1.062	<0.001
Stage				0.190
Stage II vs. Stage I	0.916	0.319	2.629	0.871
Stage III vs. Stage I	0.830	0.210	3.280	0.790
Stage IV vs. Stage I	1.778	0.431	7.330	0.426
T grade				0.260
T2 vs. T1	4.571	0.588	35.547	0.146
T3 vs. T1	6.761	0.795	57.478	0.080
T4 vs. T1	5.603	0.636	49.379	0.121
N grade				0.079
N1 vs. N0	1.236	0.583	2.621	0.580
N2 vs. N0	1.823	0.734	4.523	0.196
N3 vs. N0	2.517	1.016	6.236	0.046
M grade	1.788	0.761	4.206	0.183
DRGS	0.972	0.946	0.998	0.038

*HR, hazard ratio; CI, confidence interval.

### Molecular Characteristics of Different 15-DNA Repair Gene Signature Score Groups

**FIGURE 6:**
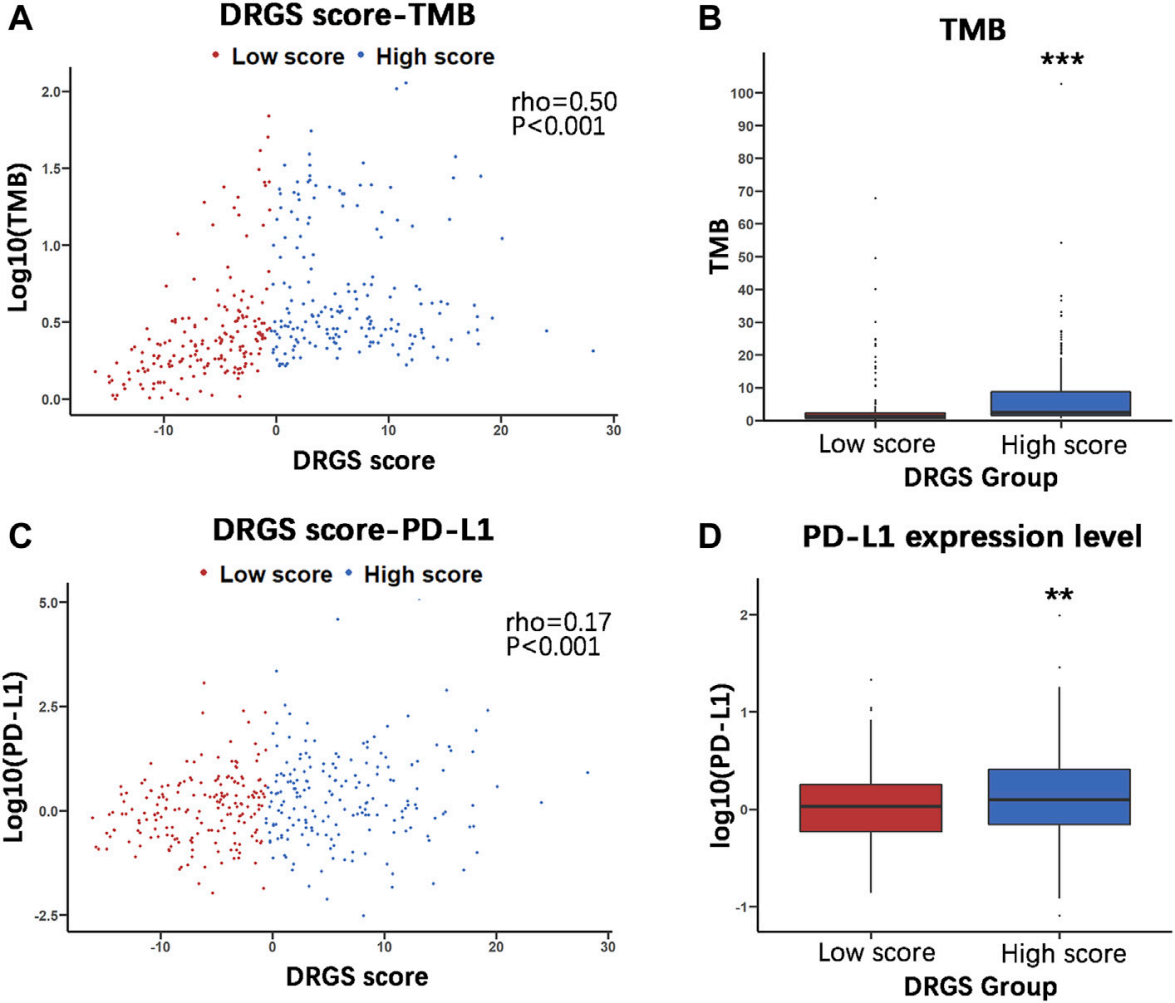
Molecular characteristics of the two DRGS score groups (High score *n* = 183, Low score *n* = 182). **(A)** and **(C)** The Spearman’s correlation was performed to analyze the relationship between the DRGS score and two important immunotherapy biomarkers, TMB and PD-L1; **(B)** and **(D)** The Mann Whitney U test was used to compare TMB and PD-L1 expression in high and low score groups in TCGA-STAD gastric cancer samples. (*p < 0.05, ** *p* < 0.01, *** *p* < 0.001).

To identify the relationship between TMB and the DRGS score, we analyzed the somatic mutation data of 365 gastric adenocarcinoma samples from TCGA-STAD (Supplementary Table S7). The result indicated that the DRGS score was positively associated with TMB (rho = 0.50, *p* < 0.001, Figure 6A). TMB of samples in DRGS high score group (*n* = 183) was significantly higher than that in DRGS low score group (*n* = 182) (*p* < 0.001, Mann-Whitney U test, Figure 6B). All mutation events were divided into different categories, among which missense mutations accounted for the largest proportion in the classification of variation (Supplementary Figure S2A, B in the Supplementary Material), single nucleotide polymorphisms (SNPs) occurred much more frequently than insertion or deletion, and C > T was the most general of single nucleotide variants (SNV) in all TCGA-STAD samples. Notably, median variant per sample of DRGS high score group (121) was higher than that of DRGS low score group (59.5). Furthermore, mutation events of the top 10 mutated genes in each sample were shown in the waterfall plot. Amongst DRGS high score group, the mutation frequency of TTN was the highest (59%), followed by TP53 (54%) and MUC16 (39%, Supplementary Figure S2B). Amongst DRGS low score group, the highest mutation frequency gene was TP53 (34%), followed by TTN (33%) and MUC16 (21%, Supplementary Figure S2A).

PD-L1 expression is another important biomarker of susceptibility to PD-L1 blockade. We found that PD-L1 expression in DRGS high score group (*n* = 183) was significantly higher than in DRGS low score group (*n* = 182) (*p* = 0.002, Figure 6D, Supplementary Table S7). The DRGS score is positively correlated to PD-L1 expression (rho = 0.17, *p* < 0.001, Figure 6C).

### Benefits of Immunotherapy With Programmed Cell Death Ligand 1 Blockers in Different 15-DNA Repair Gene Signature Score Groups

**FIGURE 7:**
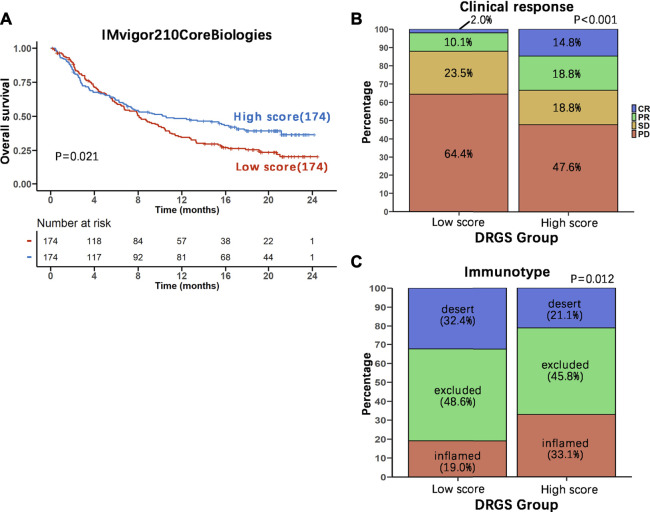
Benefits of immunotherapy with PD-L1 blockers in the two DRGS score groups. **(A)** The 348 patients in IMvigor210CoreBiologies were equally divided into two groups based on their DRGS scores. Kaplan-Meier analysis of overall survival in the two groups was performed and the survival curves were compared using the log-rank test (High score *n* = 174, Low score *n* = 174); **(B)** Comparison of the clinical response to immunotherapy between the two DRGS groups (High score *n* = 149, Low score *n* = 149); **(C)** Comparison of the three immunotypes between the two DRGS groups of patients using the Chi-square test (High score *n* = 142, Low score *n* = 142).

In view of the association between our score system and the immune microenvironment of the tumor, as described above, we tested the ability of the DRGS score system to predict the response of patients to immunotherapy. This analysis was based on the IMvigor210CoreBiologies cohort (*n* = 348), a large phase 2 trial investigating the clinical activity of PD-L1 blockade with atezolizumab in metastatic urothelial carcinoma (mUC) (Gibney et al., 2016) (Supplementary Table S8). We found that DRGS high score patients (*n* = 174) exhibited prominent prolonged overall survival (*p* = 0.021, Figure 7A). In addition, 298 patients in this cohort showed varying degrees of response to anti-PD-L1 blockers. DRGS high score patients (*n* = 149) comprised 14.8% complete response (CR), 18.8% partial response (PR), 18.8% stable disease (SD) and 47.6% progressive disease (PD), while DRGS low score patients (*n* = 149) comprised 2.0% CR, 10.1% PR, 23.5% SD and 64.4% PD. The Chi-squared test performed between DRGS high and low score groups also showed significantly better therapeutic outcomes in DRGS high score patients than the low score patients (*p* < 0.001, Figure 7B).

We also analyzed the three immune subtypes (immune inflamed, immune excluded and immune desert) of IMvigor210CoreBiologies in two DRGS group patients. DRGS high score patients (*n* = 142) comprised 33.1% inflamed, 45.8% excluded and 21.1% desert, while DRGS low score patients (*n* = 142) comprised 19.0% inflamed, 48.6% excluded and 32.4% desert. Therefore, the DRGS high score patients had a significantly higher percentage of “immune inflamed” tumors and a lower percentage of “immune desert” and “immune excluded” tumors compared to the low score patients (*p* = 0.012, Chi-square test, Figure 7C).

### 15-DNA Repair Gene Signature Score and its Potential Chemotherapeutic Value

**FIGURE 8:**
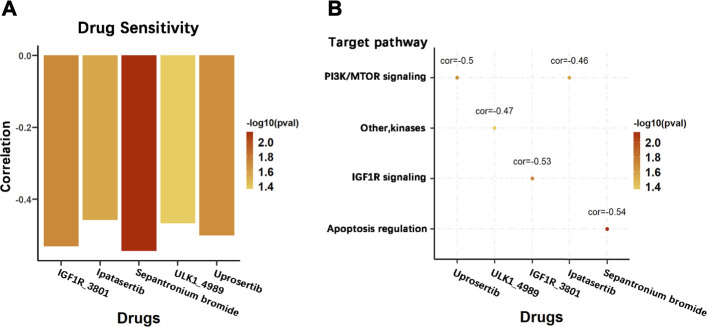
Potential therapeutic value of the DRGS score in chemotherapy. **(A)** Pearson correlation analysis was used to calculate the correlation between the sensitivity level of different chemotherapeutic agents and the DRGS score of 23 types of gastric cancer cells; **(B)** Signalling pathways targeted by the drugs that are correlated with the DRGS score.

To explore the effect of the DRGS score system on drug response in chemotherapy, we evaluated the association between the DRGS score and the response to drugs in gastric cancer cell lines (*n* = 23) in the Genomics of Drug Sensitivity in Cancer (GDSC) database. We identified 5 significantly correlated pairs between the DRGS score and drug sensitivity, all of which demonstrated drug resistance correlated with the DRGS score, including insulin-like growth factor receptor IGF1R_3801 (Rs = −0.53, *p* = 0.019), AKT inhibitor ipatasertib (Rs = −0.46, *p* = 0.028), surviving inhibitor sepantronium bromide (Rs = −0.54, *p* = 0.007), ULK1 protein kinase ULK1_4989 (Rs = −0.47, *p* = 0.043) and AKT inhibitor uprosertib (Rs = −0.50, *p* = 0.021) (Figure 8A). In addition, we found that these drugs mostly target PI3K/MTOR, IGF1R and apoptosis regulation signaling pathways (Figure 8B).

### Immunohistochemistry Verification of Expression of Genes in 15-DNA Repair Gene Signature and Relationship With Programmed Cell Death Ligand 1 Expression

**FIGURE 9:**
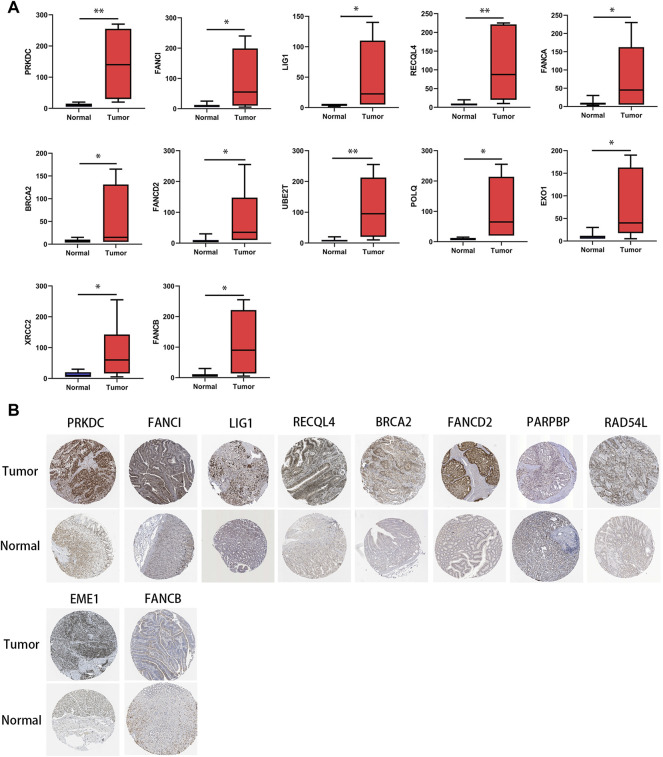
Protein level of genes in the DRGS in gastric cancer tissues and normal tissues. **(A)** IHC (Tumor *n* = 10, Normal *n* = 10); (*p < 0.05, ** *p* < 0.01). **(B)** HPA database.

Through IHC analysis, we found in gastric tumor tissues (*n* = 10) that the protein expression of most of the genes in the DRGS, except for PARPBP, EME1, and RAD54L, was significantly increased (Figure 9A). In the HPA data, compared with normal tissues, the expression of DRGS except FANCA, UBE2T, POLQ, EXO1, and XRCC2 (They were not included) was remarkably upregulated (Figure 9B).

**FIGURE 10:**
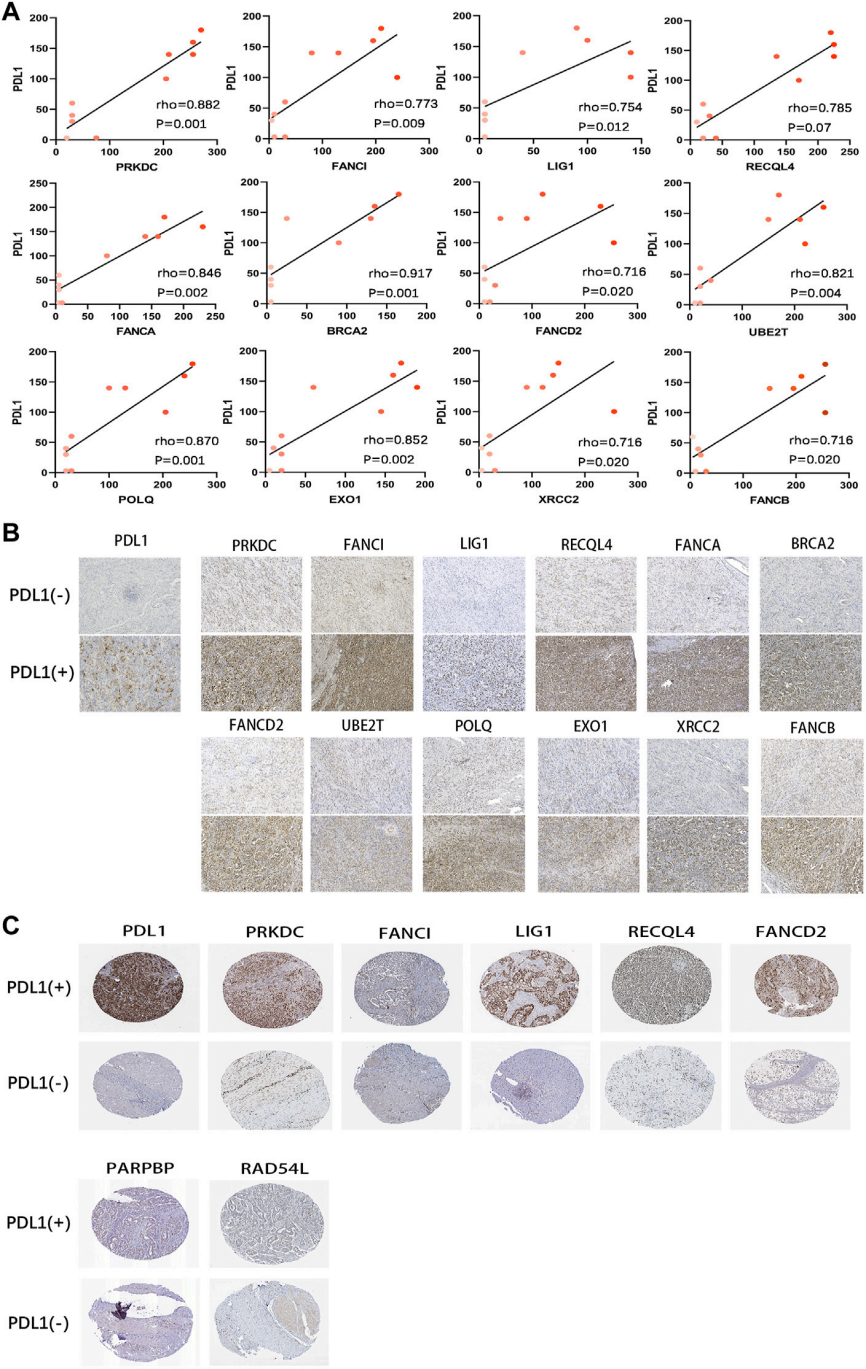
Correlation of protein expression between PD-L1 and DRGS. **(A)** The Spearman’s correlation was performed to analyze the relationship between PD-L1 and DRGS in IHC gastric cancer samples (*n* = 10). The color depth of the points represents the IHC score of DRGS; **(B)** Protein expression (IHC) of DRGS in PD-L1 (−) patients and PD-L1 (+) patients; **(C)** Data from the HPA database.

We also analysed the relationship between the DRGS and PD-L1 protein expression. As shown in Figures 10A,B, based on IHC analysis (*n* = 10), the protein expression of the genes in the DRGS, except for PARPBP, EME1, and RAD54L, was positively correlated to PD-L1 expression (rho>0.7, *p* < 0.05, Figures 10A,B). In the HPA data, the protein level of PRKDC, FANCI, LIG1, RECQL4, FANCA, PARPBP, RAD54L, and FANCD2 (The rest of the 15 genes were not included) was positively related to that of PD-L1 (Figure 10C). Thus, the above results verified the results obtained from bioinformatics analysis.

## Discussion

In the emerging immunotherapy strategies such as the ICB therapy in various types of cancer, the interaction of tumor DNA damage and repair landscape with patient immune system and related therapy has recently been revealed as a complex biological process (Fridman et al., 2012; Gentles et al., 2015; Mouw et al., 2017; Bever and Le, 2018; Joshi and Badgwell, 2021; Zhang et al., 2021). Nevertheless, many prognostic and predictive biomarkers are being evaluated clinically to identify criteria for establishing customized therapeutic approaches. This has been verified in cancers with MMR deficiency, which leads to microsatellite instability (MSI) phenotype, in which ICIs were found to be more efficient (Le et al., 2017; Bever and Le, 2018; Joshi and Badgwell, 2021).

In this study, based on the known association of DNA damage and repair with immunotherapy response, we identified a 15-DNA repair gene signature (DRGS) using the gene expression data in TCGA-STAD and GSE 30727 datasets and developed the scoring system with the median score as the cut-point to divide gastric cancer patients into high and low score subtypes. We used the IMvigor210CoreBiologies cohort to assess the ability of the DRGS score to predict patient response to ICB therapy. The results demonstrated that DRGS high score patients showed significantly better therapeutic outcomes compared to the low score patients (*p* < 0.001), suggesting that the DRGS is a promising predictor of patient response to immune therapies.

The 15 genes in DRGS were screened from those that are significantly differentially expressed between tumor and normal tissues in the TCGA-STAD and GSE30727 datasets. Of the DRGS genes, six are associated with tolerance and repair of DNA crosslinks, including six genes from the FA pathway: FANCI, FANCA, FANCD2, FANCB, BRCA2 (FANCD1), and XRCC2. This fact may indicate a damage response to DNA crosslinks formed in the gastric cancer, although no information is available on a possibly higher level of such lesions in this disease. The FA pathway has also been linked to cancer susceptibility, with either sensitivity or resistance to chemotherapeutic agents. Also, four genes in the DRGS are in the HR pathway for the repair of DNA double-strand breaks, i.e., XRCC2, EME1, RAD54L, and BRCA2. This pathway is among the most well-studied of DNA repair defects in tumor immunotherapy response (Strickland et al., 2016; Mouw et al., 2017). It has been suggested that HR-deficient (HRD) tumors may possess enhanced immunogenicity, and thus, become more susceptible to checkpoint inhibitors (van Wilpe et al., 2021). The HPA database and IHC analysis were used to successfully verify the differential expression of these genes in human gastric cancer tissues. It should also be noted that in the DRGS high score group, the high expression level of the DNA repair genes does not mean an increased cellular repair capacity, rather, it could well be a damage response following the increased levels of DNA damage inside tumor cells. Therefore, a better understanding of the DNA damage in the tumor will help to know the underlying mechanism of the DRGS as a biomarker for selection patients for immunotherapy. In addition, changes in gene expression levels and protein levels do not correlate well in most of the cases, mainly due to the regulation control at different levels.

It is known that the tumor immune microenvironment has great implications in gastric cancer progression and susceptibility to immunotherapy (Lazăr et al., 2018). Given that a multifaceted condition may affect gastric cancer primed for response to immunotherapy, we analyzed several important factors or potential immunotherapy response biomarkers in relation to the DRGS high and low score groups using TCGA-STAD, including tumor-infiltrating lymphocytes, immune subtypes, TMB, PD-L1 expression and other histologic and molecular classifications (detailed in Results). In TCGA-STAD samples analyzed by ImmuCellAI, we found that anti-tumor cells including γδ T cells, neutrophils and Th1 cells were more abundant in the DRGS high score samples, while several types of immunosuppressive or tumor enhancement cells were more abundant in the low-score patients. Next, we analyzed the somatic mutation and PD-L1 expression data in TCGA-STAD and found that both were positively associated with the score, suggesting that DRGS high score patients are more likely to respond to immunotherapy. We also analyzed the three immune subtypes of IMvigor210CoreBiologies and found that the DRGS high score patients were associated with a significantly higher percentage of “immune inflamed” subtype, which has been shown to be infiltrated by a number of subtypes of immune cells (Chen and Mellman, 2017). Finally, the DRGS high score group was shown to have a higher percentage of MSI-H phenotype in the TCGA subtypes. MSI-H is characterized by a better survival (Yamamoto et al., 1999) and a high number of microsatellite mutations (Yoon et al., 2013). Recent clinical trials have confirmed that MSI-positive gastric tumors are sensitive to ICB therapy (Le et al., 2015). In summary, our DRGS high-score gastric cancer patients are characterized by “immune inflamed”, “intestinal”, and “MSI-H” phenotypes, which indicate an abundant immune cell infiltration, high expression of PD-L1, and high number of microsatellite mutations. All of which are associated with higher sensitivity and better response to immunotherapy. The verification of correlation between DRGS and PD-L1 protein expression with HPA database and our IHC results were consistent with the above observations.

There were also limitations in this work. We used the data from a different type of tumor (IMvigor210CoreBiologies cohort on locally advanced or metastatic urothelial bladder cancer) to evaluate the ability of the DRGS score to predict PD-L1 blockade-based immunotherapy response, because of a lack of such a dataset in gastric cancer. Future studies will use more data from the gastric cancer patients, including those from our own hospital.

## Conclusion

The DRGS is a promising predictor of patient response to immune checkpoint inhibitor (ICI)-based therapy. Further studies are needed to evaluate the clinical utility of the DRGS for tailoring immunotherapy.

## Data Availability

The data used for analysis in this study are available from TCGA-STAD, GEO, GDSC, CPTAC, HPA and IMvigor210CoreBiologies databases. All the original codes have been uploaded to Zenodo website. The link is https://doi.org/10.5281/zenodo.6466144.

## Supplementary Material

Supplementary Figure S1 PCA analysis was used as a dimensionality reduction method to obtain the first-dimension correlation coefficients of 15 repair genes in IMvigor210CoreBiologies, GSE26901, GSE15459, GSE26899, and gastric cancer cell lines (from A to E).

Supplementary Figure S2 Overview of TCGA-STAD mutations. Statistics and visual display of mutation type, SNV classification, TMB and mutation classification in DRGS low score subtype **(A)** versus high score subtype **(B)** samples. The Waterfall plot of the top 10 mutant genes, which classifies the mutations of each gene in each sample.

## Glossary

BER: base excision repair
CIN: chromosomal instability
CR: complete response
DRGS: 15-DNA repair gene signature
DSB: double-strand break
GDSC: Genomics of Drug Sensitivity in Cancer
GS: genomically stable
HR: homologous recombination
HRD: homologous recombination deficiency
ICB: immune checkpoint blockade
ICI: immune checkpoint inhibitors
IHC: Immunohistochemistry
MMR: mismatch repair
MSI: microsatellite instability
MSI-H: microsatellite instability-high
MSI-L: microsatellite instability-low
MSS: Microsatelite Instability-Stable
mUC: metastatic urothelial carcinoma
NER: nucleotide excision repair
NHEJ: non-homologous end joining
OS: overall survival
PCA: principal component analysis
PD: progressive disease
PD-1: programmed cell death protein 1
PD-L1: programmed cell death ligand 1
PR: partial response
RFS: relapse-free survival
SD: stable disease
SNV: single nucleotide variants
TCGA-STAD: The Cancer Genome Atlas Stomach Adenocarcinoma
TMB: tumor mutation burden

## References

[B1] Abbott B. P. Abbott R. Abbott T. D. Abernathy M. R. Acernese F. Ackley K. (2016). Observation of Gravitational Waves from a Binary Black Hole Merger. Phys. Rev. Lett. 116 (6), 061102. 10.1103/PhysRevLett.116.061102 26918975

[B2] Balar A. V. Galsky M. D. Rosenberg J. E. Powles T. Petrylak D. P. Bellmunt J. (2017). Atezolizumab as First-Line Treatment in Cisplatin-Ineligible Patients with Locally Advanced and Metastatic Urothelial Carcinoma: a Single-Arm, Multicentre, Phase 2 Trial. Lancet 389 (10064), 67–76. 10.1016/s0140-6736(16)32455-2 27939400 PMC5568632

[B3] Bever K. M. Le D. T. (2018). DNA Repair Defects and Implications for Immunotherapy. J. Clin. Invest. 128 (10), 4236–4242. 10.1172/jci122010 30272580 PMC6159999

[B4] Chalmers Z. R. Connelly C. F. Fabrizio D. Gay L. Ali S. M. Ennis R. (2017). Analysis of 100,000 Human Cancer Genomes Reveals the Landscape of Tumor Mutational Burden. Genome Med. 9 (1), 34. 10.1186/s13073-017-0424-2 28420421 PMC5395719

[B5] Chao J. Fuchs C. S. Shitara K. Tabernero J. Muro K. Van Cutsem E. (2020). Pembrolizumab (Pembro) in Microsatellite Instability-High (MSI-H) Advanced Gastric/gastroesophageal Junction (G/GEJ) Cancer by Line of Therapy. Jco 38 (4_Suppl. l), 430. 10.1200/jco.2020.38.4_suppl.430

[B6] Chen D. S. Mellman I. (2017). Elements of Cancer Immunity and the Cancer-Immune Set Point. Nature 541 (7637), 321–330. 10.1038/nature21349 28102259

[B7] Fridman W. H. Pagès F. Sautès-Fridman C. Galon J. (2012). The Immune Contexture in Human Tumours: Impact on Clinical Outcome. Nat. Rev. Cancer 12 (4), 298–306. 10.1038/nrc3245 22419253

[B8] Friedberg E. C. Walker G. C. Si Ede W. (2015). A History of the DNA Repair and Mutagenesis Field. Dna Repair 33 (2), 35–42. 10.1016/j.dnarep.2015.06.007 26151545

[B9] Fuchs C. S. Özgüroğlu M. Bang Y.-J. Di Bartolomeo M. Mandala M. Ryu M.-H. (2021). Pembrolizumab versus Paclitaxel for Previously Treated PD-L1-Positive Advanced Gastric or Gastroesophageal Junction Cancer: 2-year Update of the Randomized Phase 3 KEYNOTE-061 Trial. Gastric Cancer 25, 197–206. 10.1007/s10120-021-01227-z 34468869 PMC8732941

[B10] Gentles A. J. Newman A. M. Liu C. L. Bratman S. V. Feng W. Kim D. (2015). The Prognostic Landscape of Genes and Infiltrating Immune Cells across Human Cancers. Nat. Med. 21 (8), 938–945. 10.1038/nm.3909 26193342 PMC4852857

[B11] Gibney G. T. Weiner L. M. Atkins M. B. (2016). Predictive Biomarkers for Checkpoint Inhibitor-Based Immunotherapy. Lancet Oncol. 17 (12), e542–e551. 10.1016/s1470-2045(16)30406-5 27924752 PMC5702534

[B12] Hang B. (2004). Repair of Exocyclic DNA Adducts: Rings of Complexity. Bioessays 26 (11), 1195–1208. 10.1002/bies.20130 15499577

[B13] Harada K. Baba H. Ajani J. A. (2018). Recent Trend in Gastric Cancer Treatment in the USA. Jcmt 4, 18. 10.20517/2394-4722.2017.74 34113719 PMC8188734

[B14] Helleday T. Petermann E. Lundin C. Hodgson B. Sharma R. A. (2008). DNA Repair Pathways as Targets for Cancer Therapy. Nat. Rev. Cancer 8 (3), 193–204. 10.1038/nrc2342 18256616

[B15] Joshi S. S. Badgwell B. D. (2021). Current Treatment and Recent Progress in Gastric Cancer. CA A Cancer J. Clin. 71 (3), 264–279. 10.3322/caac.21657 PMC9927927 33592120

[B16] Lazăr D. C. Avram M. F. Romoșan I. Cornianu M. Tăban S. Goldiș A. (2018). Prognostic Significance of Tumor Immune Microenvironment and Immunotherapy: Novel Insights and Future Perspectives in Gastric Cancer. Wjg 24 (32), 3583–3616. 10.3748/wjg.v24.i32.3583 30166856 PMC6113718

[B17] Le D. T. Durham J. N. Smith K. N. Wang H. Bartlett B. R. Aulakh L. K. (2017). Mismatch Repair Deficiency Predicts Response of Solid Tumors to PD-1 Blockade. Science 357 (6349), 409–413. 10.1126/science.aan6733 28596308 PMC5576142

[B18] Le D. T. Uram J. N. Wang H. Bartlett B. R. Kemberling H. Eyring A. D. (2015). PD-1 Blockade in Tumors with Mismatch-Repair Deficiency. N. Engl. J. Med. 372 (26), 2509–2520. 10.1056/NEJMoa1500596 26028255 PMC4481136

[B19] Mayakonda A. Lin D.-C. Assenov Y. Plass C. Koeffler H. P. (2018). Maftools: Efficient and Comprehensive Analysis of Somatic Variants in Cancer. Genome Res. 28 (11), 1747–1756. 10.1101/gr.239244.118 30341162 PMC6211645

[B20] Miao Y. R. Zhang Q. Lei Q. Luo M. Xie G. Y. Wang H. (2020). ImmuCellAI: A Unique Method for Comprehensive T‐Cell Subsets Abundance Prediction and its Application in Cancer Immunotherapy. Adv. Sci. 7 (7), 1902880. 10.1002/advs.201902880 PMC7141005 32274301

[B21] Moehler M. Shitara K. Garrido M. Salman P. Shen L. Wyrwicz L. (2020). LBA6_PR Nivolumab (Nivo) Plus Chemotherapy (Chemo) versus Chemo as First-Line (1L) Treatment for Advanced Gastric Cancer/gastroesophageal Junction Cancer (GC/GEJC)/esophageal Adenocarcinoma (EAC): First Results of the CheckMate 649 Study - ScienceDirect. Ann. Oncol. 31. 10.1016/j.annonc.2020.08.2296

[B22] Mouw K. W. Goldberg M. S. Konstantinopoulos P. A. D'Andrea A. D. (2017). DNA Damage and Repair Biomarkers of Immunotherapy Response. Cancer Discov. 7 (7), 675–693. 10.1158/2159-8290.Cd-17-0226 28630051 PMC5659200

[B23] Rhyu M. G. Park W. S. Meltzer S. J. (1994). Microsatellite Instability Occurs Frequently in Human Gastric Carcinoma. Oncogene 9 (1), 29–32. 8302591

[B24] Rodríguez A. D’Andrea A. (2017). Fanconi Anemia Pathway. Curr. Biol. 27 (18), R986–r988. 10.1016/j.cub.2017.07.043 28950089

[B25] Strickland K. C. Howitt B. E. Shukla S. A. Rodig S. Ritterhouse L. L. Liu J. F. (2016). Association and Prognostic Significance of BRCA1/2-Mutation Status with Neoantigen Load, Number of Tumor-Infiltrating Lymphocytes and Expression of PD-1/pd-L1 in High Grade Serous Ovarian Cancer. Oncotarget 7 (12), 13587–13598. 10.18632/oncotarget.7277 26871470 PMC4924663

[B26] The Cancer Genome Atlas Research Network (2014). Comprehensive Molecular Characterization of Gastric Adenocarcinoma. Nature 513 (7517), 202–209. 10.1038/nature13480 25079317 PMC4170219

[B27] van Wilpe S. Tolmeijer S. H. Koornstra R. H. T. de Vries I. J. M. Gerritsen W. R. Ligtenberg M. (2021). Homologous Recombination Repair Deficiency and Implications for Tumor Immunogenicity. Cancers 13 (9), 2249. 10.3390/cancers13092249 34067105 PMC8124836

[B28] Wood R. D. Mitchell M. Lindahl T. (2005). Human DNA Repair Genes, 2005. Mutat. Research/Fundamental Mol. Mech. Mutagen. 577, 275–283. 10.1016/j.mrfmmm.2005.03.007 15922366

[B29] Yamamoto H. Perezpiteira J. Yoshida T. Terada M. Itoh F. Imai K. (1999). Gastric Cancers of the Microsatellite Mutator Phenotype Display Characteristic Genetic and Clinical Features☆, ☆☆. Gastroenterology 116 (6), 1348–1357. 10.1016/s0016-5085(99)70499-3 10348818

[B30] Yoon K. Lee S. Han T.-S. Moon S. Y. Yun S. M. Kong S.-H. (2013). Comprehensive Genome- and Transcriptome-wide Analyses of Mutations Associated with Microsatellite Instability in Korean Gastric Cancers. Genome Res. 23 (7), 1109–1117. 10.1101/gr.145706.112 23737375 PMC3698504

[B31] Yuan B. Jiang C. Chen L. Cui J. Chen M. Zhang S. (2021). A 15-DNA Repair Gene Prognostic Signature for Immunotherapy in Gastric Cancer. Preprints 100297, 2021. 10.20944/preprints202110.0297.v1

[B32] Zhang C. Li D. Yu R. Li C. Song Y. Chen X. (2021). Immune Landscape of Gastric Carcinoma Tumor Microenvironment Identifies a Peritoneal Relapse Relevant Immune Signature. Front. Immunol. 12, 651033. 10.3389/fimmu.2021.651033 34054812 PMC8155484

