# Supplementary figures and images for: A Novel DNA Repair Gene Signature for Immune Checkpoint Inhibitor-Based Therapy in Gastric Cancer
Source: Front Cell Dev Biol. 2022 May 23;10:893546. doi: 10.3389/fcell.2022.893546 (PMC9168368; doi:10.3389/fcell.2022.893546)

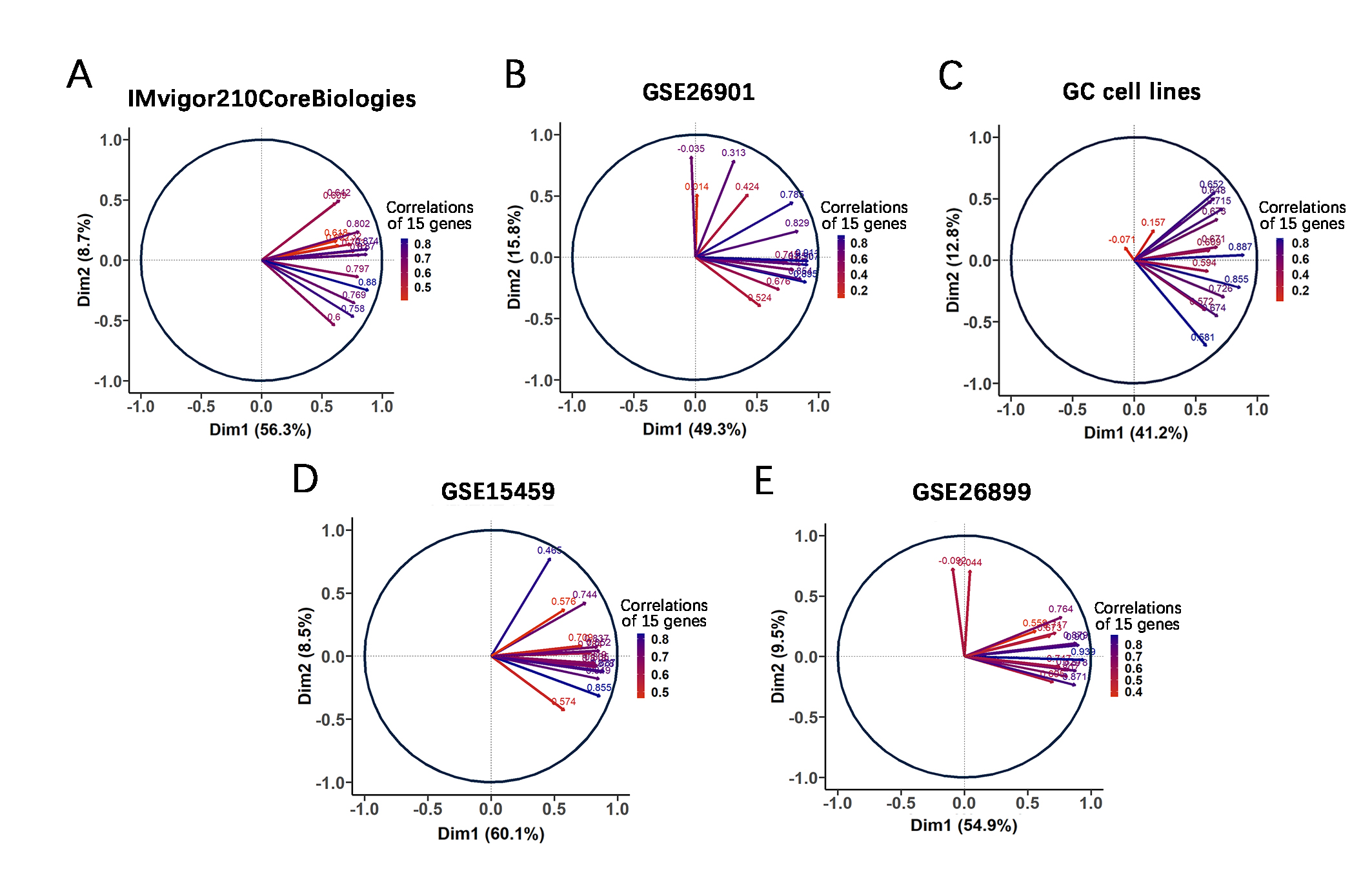

Supplement: Supplementary file 3 [file Image1.JPEG]

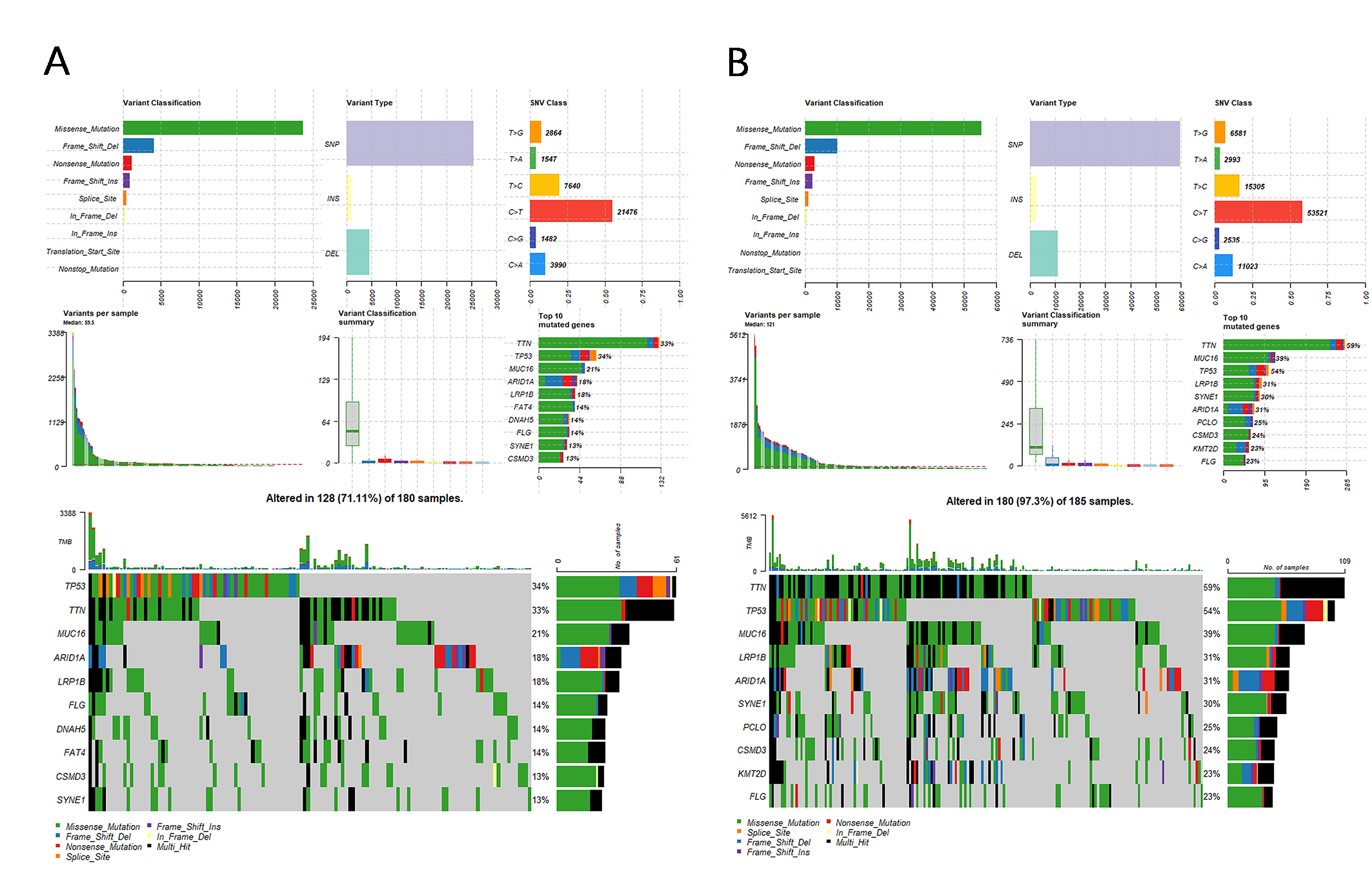

Supplement: Supplementary file 4 [file Image2.JPEG]
